# Differentially-Expressed Pseudogenes in HIV-1 Infection

**DOI:** 10.3390/v7102869

**Published:** 2015-09-29

**Authors:** Aditi Gupta, C. Titus Brown, Yong-Hui Zheng, Christoph Adami

**Affiliations:** 1 Department of Microbiology and Molecular Genetics, Michigan State University, 567 Wilson Road, East Lansing, MI 48824, USA; ctbrown@ucdavis.edu (C.T.B.); zhengyo@msu.edu (Y.-H.Z.); adami@msu.edu (C.A.); 2 BEACON Center for the Study of Evolution in Action, Michigan State University, 567 Wilson Road, East Lansing, MI 48824, USA; 3 Department of Computer Science and Engineering, Michigan State University, 428 S. Shaw Lane, East Lansing, MI 48824, USA; 4 Department of Physics and Astronomy, Michigan State University, 567 Wilson Road, East Lansing, MI 48824, USA

**Keywords:** pseudogenes, HIV-1, differential gene expression, transcriptome, RNASeq

## Abstract

Not all pseudogenes are transcriptionally silent as previously thought. Pseudogene transcripts, although not translated, contribute to the non-coding RNA pool of the cell that regulates the expression of other genes. Pseudogene transcripts can also directly compete with the parent gene transcripts for mRNA stability and other cell factors, modulating their expression levels. Tissue-specific and cancer-specific differential expression of these “functional” pseudogenes has been reported. To ascertain potential pseudogene:gene interactions in HIV-1 infection, we analyzed transcriptomes from infected and uninfected T-cells and found that 21 pseudogenes are differentially expressed in HIV-1 infection. This is interesting because parent genes of one-third of these differentially-expressed pseudogenes are implicated in HIV-1 life cycle, and parent genes of half of these pseudogenes are involved in different viral infections. Our bioinformatics analysis identifies candidate pseudogene:gene interactions that may be of significance in HIV-1 infection. Experimental validation of these interactions would establish that retroviruses exploit this newly-discovered layer of host gene expression regulation for their own benefit.

## 1. Introduction

Pseudogenes are considered relics of once-functional genes that have lost the ability to code for proteins. They arise due to mutations, frame-shifts and gene duplication events, where the duplicated gene loses transcriptional elements (like promoters and enhancers) necessary for gene expression [1]. Irrespective of how they are created, pseudogenes are traditionally assumed to be transcriptionally silent. However, microarray studies show that 2%–20% of pseudogenes are quantifiably expressed [1,2,3]. Tissue-specific expression of pseudogenes hints at their possible functional importance in those tissues [4,5]. Pseudogenes are also differentially expressed in diseases, notably cancer [6,7,8,9,10,11,12].

We are only beginning to understand the implications of pseudogene expression for cellular well-being. Pseudogenes can regulate the expression of parent genes by the following mechanisms [1]: (i) antisense pseudogene transcripts can pair with a sense parent transcript, altering the parent gene mRNA levels [13,14]; (ii) pseudogene transcripts are sometimes processed to produce small interfering RNAs that silence the parent gene [15,16]; and (iii) pseudogene transcripts can form competitive endogenous RNA (ceRNA) that competes with the parent gene transcripts for the shared pool of mRNA stability factors and miRNA [17,18,19,20]. Tumor suppressor gene PTEN, for example, is post-transcriptionally regulated by microRNAs that target transcripts of both PTEN and its pseudogene PTENP1. Yet, overexpression of the PTENP1 3${}^{\prime }$ UTR recovers PTEN mRNA levels, highlighting the functional role of pseudogene PTENP1 transcripts in regulating parent-gene mRNA abundance levels [20]. Moreover, transcriptional networks are reported to be cross-regulated with ceRNA networks, suggesting that pseudogene-derived transcripts play an integral role in regulating cellular gene expression [21]. Pseudogene-derived endogenous siRNA can also regulate the expression of genes other than its parent gene [12]. In cancer, pseudogenes have been shown to affect the expression of non-parent genes as well, such as tumor suppressor gene MGA, when integrated into their promoter regions [9]. By contributing substantially to the small-RNA pool of a cell, pseudogenes may regulate the expression of their parent gene, as well as other genes.

The majority of pseudogenes in humans are “processed”, *i.e.*, they are created when a reverse-transcribed mRNA is re-integrated into the genome, often on chromosomes different from the parent gene [1,22,23]. Thus, these “processed” pseudogenes lack introns and contain poly-A tails. They also have direct repeats at the 3${}^{\prime }$ and 5${}^{\prime }$ ends that likely participate in the genome re-integration step [1,22,24]. However, how are these processed mRNA transcripts re-integrated back into the genome? Some long interspersed elements (LINEs) code for reverse transcriptase that makes the cDNA copy of mRNAs, possibly contributing to the generation of the processed pseudogenes [9,25,26]. Thus, retroviruses, such as HIV-1, by carrying viral reverse transcriptase and integrase to the infected cells, may create new pseudogenes and alter the expression of existing pseudogenes [27,28]. Carlton *et al.* find evidence for the generation of a processed pseudogene during retroviral infection and speculate that viral reverse transcriptase is involved in the process [28]. It is thus likely that pseudogene:gene interactions and pseudogene-derived endogenous non-coding RNA constitute another dimension of virus-host interactions. The impact of non-coding RNAs on HIV-1 replication and pathogenesis has already been discussed elsewhere [29], such as the role of host-induced antiviral microRNA in limiting the progression of viral infection [30].

In this study, we investigate differential expression of pseudogenes in HIV-1 infection by comparing the transcriptomes of infected and uninfected T-cells seven days post-infection. By identifying parent and other genes that show sequence similarity to these pseudogenes, we predict candidate pseudogene:gene interactions that may be relevant to HIV-1 infection. We further analyze publicly-available transcriptomic data collected at 12 h and 24 h post-HIV-1 infection to identify time-course changes in the expression of pseudogenes and their parent genes. Our results demonstrate that pseudogenes are active participants in host-pathogen interactions, and their role in modulating host gene expression should be investigated further.

## 2. Materials and Methods

### 2.1. Cells

The human T-cell line H9 was obtained from the NIH AIDS Research and Reference Reagent Program (Catalog Number 87) [31] and propagated in RPMI 1640 medium (Gibco) supplemented with 10% fetal bovine serum (HyClone) and 1% 100X Antibiotic-Antimycotic (Gibco). The NL4-3 strain of HIV-1 was obtained from the NIH AIDS Research and Reference Reagent Program (Catalog Number 9489), and the virus was prepared by transfection of 293T cells. 293T cells were purchased from ATCC and propagated in Dulbecco’s Modified Eagle’s Medium (DMEM) with 10% bovine calf serum (HyClone).

### 2.2. HIV-1 Infection

To generate transcriptomic data, we infected $5\times 10^{6}$ H9 T-cells with 300 ng $p24^{Gag}$ NL43 strain of HIV-1 using a spinfection protocol (multiplicity of infection > 1): infected cells were incubated at 37 °C for 15 min and then centrifuged at 1200 rpm for 2 h at 33 °C, followed by a further half-hour incubation at 37 °C and three rounds of washing with RPMI. The infected cells were then replenished with fresh medium and incubated at 37 °C for seven days. Then, $3\times 10^{6}$ uninfected H9 T-cells were used as the control and incubated for 7 days in conditions identical to the infected T-cells (37 °C, 5% $CO_{2}$). Cellular RNA from infected and uninfected cells was extracted for sequencing 7 days post-infection using Qiagen’s RNeasy Mini Kit.

### 2.3. RNA Sequencing

Paired-end (150 bp × 2) RNASeq data were generated using the Illumina HiSeq sequencing platform. The uninfected sample generated 82 million paired-end reads (24 Gbp) with a mean fragment size of 377 bp, and the infected sample generated 76 million reads (23 Gbp) with a mean fragment size of 361 bp. Illumina adaptors were removed using Trimmomatic Version 0.3, and low-quality reads were dropped using fastq_quality_filter [32]. Ribosomal RNA was depleted, and poly-A RNA was selected prior to sequencing. The sequence data generated were of high quality. The average quality score for the forward reads was >34, and that for the reverse reads was >33 (a Q-score of 30 is considered optimal). The transcriptomics data are available at the NCBI’s Gene Expression Omnibus (GEO) database, via Accession Number GSE70785.

### 2.4. Pipeline to Identify Differentially-Expressed Pseudogenes

The filtered reads were mapped to the human reference transcriptome using Tophat2 Version 2.0.11 [33]. The human reference genome (fasta file: Homo_sapiens.GRCh37.57.dna.toplevel.fa.gz, indexed using Bowtie2 Version 2.2.1 [34]) and the most recent human gene model (Gene Transfer Format (GTF) file: Homo_sapiens.GRCh37.75.gtf) were downloaded from Ensembl. More than 90% of reads from the uninfected and the infected sample mapped to the human reference transcriptome. The mapped reads were then supplied to the Cuffdiff program of Cufflinks (Version 2.2.0) to quantify gene expression, as well as to calculate the $\log_{2}$ fold change in gene expression due to HIV-1 infection [35]. Cuffdiff reports gene expression in FPKM (fragments per kilobase of transcript per million mapped reads).

To avoid false positives due to noise in measuring very low expression levels, we limited ourselves to genes with expression >0.1 FPKM in both infected and uninfected cells, a threshold widely used for reliable detection of transcripts in RNASeq data [36,37,38]. The 16,243 mRNA transcripts fulfilling this criteria were further pruned to only those that showed at least a 4-fold increase or decrease in gene expression due to infection (*i.e.*, a $\log_{2}$ fold change in expression >2 or <−2). We focused on fold change in gene expression, because this quantity is comparable across genes with different basal expression levels. This resulted in 493 transcripts with at least a 4-fold change in expression, of which 46 are annotated as pseudogenes in Ensembl based on GENCODE (ENCyclopedia Of DNA Elements- genes and gene variants) annotations [26].

**Figure 1:**
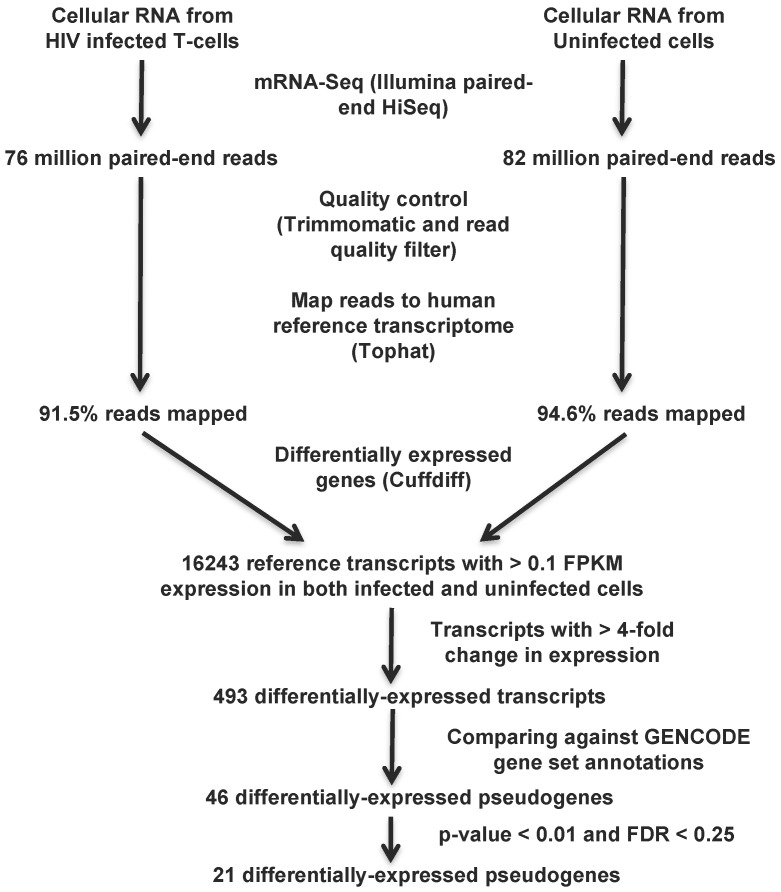
Computational pipeline to identify differentially-expressed pseudogenes from transcriptomic data. The transcriptomic data collected from HIV-1 infected and uninfected H9 T-cells 7 days post-infection were mapped to the human reference transcriptome after quality control analysis. Differentially-expressed pseudogenes were identified following several stringent filtering steps that are the accepted standard in the field: at least >4-fold change in expression, *p*-value < 0.01 and FDR < 0.25.

We then computed the *p*-values and false discovery rate (FDR) for these 46 differentially-expressed pseudogenes as follows: the 16,243 genes with expression level >0.1 FPKM in both infected and uninfected cells had an average $\log_{2}$ (fold change in expression) of −0.037 (*μ*) with a standard deviation (*σ*) of 0.819 (the log fold change distribution is normally distributed; Supplementary Figure S1). Using this *μ* and *σ*, we calculated z-scores for the pseudogene change in gene expression as z=(x−μ)/s, where *x* is the $\log_{2}$ (fold change). *p*-values for the two-sided test were determined as the cdf of the normal distribution, and the FDR (Q-value) was calculated using the *qvalue* function of R [39]. The computational pipeline to identify differentially-expressed pseudogenes is outlined in Figure 1.

### 2.5. Bioinformatics Analysis of Candidate Pseudogenes

Human proteins matching the translated pseudogene sequence were identified by BLASTX against the GENCODE/Ensembl database using a cutoff E-value of $10^{-4}$. BLASTX hits that were uncharacterized novel protein coding genes are not reported. cDNAs (transcripts/splice variants) matching pseudogene sequences were identified using BLASTN against the Ensembl cDNA database using a cutoff E-value of $10^{-4}$ and sequence identity of >90%. cDNA hits for uncharacterized genes and other pseudogenes were ignored.

### 2.6. Analyses of Publicly-Available Transcriptomic Data

We obtained transcriptomes from HIV-1-infected cells 12 h and 24 h post-infection from the Gene Expression Omnibus database (GEO ID: GSE53993) [40]. The study authors infected human T-cell line SUP-T1 (obtained from the American Type Culture Collection) with the HIV-1 strain LAI (obtained from the NIH AIDS Research and Reference Reagent Program). The authors submitted the RNASeq data to the GEO database for the following: from HIV-1-infected cells 12 h post-infection (3 replicates), mock infected cells 12 h post-infection (3 replicates; mock infection with SUP-T1 cell-conditioned medium), HIV-1-infected cells 24 h post-infection (3 replicates) and mock infected cells 24 h post-infection (2 replicates) [40]. We analyzed these RNASeq datasets as described above: quality control followed by mapping the reads to human reference transcriptome using Tophat2 and quantifying gene expression using Cuffdiff to determine the $\log_{2}$ fold change in gene expression. This generated 9 sets of gene expression comparisons at the 12-h time point (3 replicates of Mock transcriptomes compared to 3 replicates of HIV-1 infection transcriptomes: a total of 9 comparisons) and 6 sets of gene expression comparisons at the 24-h time point (2 replicates of Mock × 3 replicates of HIV-1 infection transcriptomes). These 15 transcriptomics datasets had 14–23 million paired-end reads each, with a 73%–88% overall read mapping rate to the reference human transcriptome. Supplementary Table S2 lists the $\log_{2}$ fold change in gene expression at 12 h and 24 h post-infection for the pseudogenes and their parent genes.

## 3. Results

Using a *p*-value < 0.01 as a cutoff, we identified 13 pseudogenes as significantly over-expressed and 17 as significantly under-expressed in HIV-1-infected T-cells (Table 1). Using an FDR cutoff of 0.25, as previously used for exploratory gene expression analyses [41,42], we further narrowed the number of differentially-expressed pseudogenes to 21 (eight over-expressed and 13 under-expressed; shaded rows in Table 1). Most of the over-expressed pseudogenes are processed (Table 1). We also find several uncharacterized pseudogenes as differentially expressed (pseudogene names with prefix RP followed by a number).

**Table 1:** Differentially-expressed pseudogenes in HIV-1 infection 7 days post-infection. Pseudogene expression is measured from RNASeq data as fragments per kilobase of transcript per million mapped reads (FPKM). Pseudogenes with a *p*-value < 0.01 and FDR < 0.25 are shaded and are deemed differentially expressed.

Pseudogene	Type	Expression in Uninfected T-Cells	Expression in HIV-1 Infected T-Cells	$\log_{2}$ (Fold Change)	*p*-value	Q-value
**A. Over-expressed**						
DUTP1	Processed	0.159	2.321	3.869	1.85e-06	0.0012
RP1-89D4.1	Processed	0.200	1.377	2.782	0.0006	0.0696
RP11-720N19.1	Processed	0.102	0.676	2.722	0.0007	0.0824
UBE2FP1	Processed	1.277	7.716	2.595	0.0013	0.1183
RP11-170L3.6	Processed	0.312	1.887	2.595	0.0013	0.1183
MTATP8P2	Processed	2.744	16.526	2.59	0.0013	0.1193
RP11-265N6.3	Processed	0.106	0.617	2.536	0.0017	0.1348
MTND4P15	Unprocessed	0.362	2.097	2.535	0.0017	0.1348
IFNL3P1	Unprocessed	0.128	0.632	2.305	0.0042	0.2537
RP11-44M6.3	Processed	0.255	1.235	2.275	0.0048	0.2693
CTD-2611O12.6	Processed	0.183	0.882	2.266	0.0049	0.2736
HMGB3P24	Processed	0.11	0.504	2.191	0.0065	0.3153
AOC4P	Unprocessed	0.126	0.546	2.121	0.0084	0.3688
**B. Under-expressed**						
RP11-380G5.3	Processed	1.131	0.103	−3.463	2.868e-05	0.0103
ZNF137P	Unprocessed	1.203	0.141	−3.092	0.0002	0.0353
RP11-114F3.5	Processed	0.924	0.113	−3.034	0.0003	0.0415
SCML2P2	Processed	0.955	0.119	−3.003	0.0003	0.0439
ANTXRLP1	Unprocessed	1.507	0.207	−2.865	0.0006	0.0686
HNRNPA3P6	Processed	0.966	0.138	−2.812	0.0007	0.078
RP1-224A6.8	Processed	0.903	0.129	−2.808	0.0007	0.0787
AC010733.5	Processed	3.683	0.555	−2.731	0.001	0.1026
RP11-411B10.4	Unprocessed	0.943	0.152	−2.632	0.0015	0.1284
RP11-490K7.4	Processed	2.187	0.358	−2.613	0.0017	0.1345
KLHL2P1	Unprocessed	0.692	0.113	−2.611	0.0017	0.1348
RP11-471L13.3	Processed	1.6	0.263	−2.608	0.0017	0.1348
CTD-2008A1.2	Unprocessed	1.652	0.276	−2.58	0.0019	0.1457
GUSBP2	Unprocessed	1.124	0.217	−2.375	0.0043	0.2551
RP11-1166P10.1	Unprocessed	0.766	0.154	−2.311	0.0055	0.2849
AKR7A2P1	Processed	0.46	0.1	−2.199	0.0083	0.3665
ADAMTS7P4	Unprocessed	1.06	0.234	−2.182	0.0088	0.3744

### 3.1. Pseudogenes Derived from Over-Expressed Genes

Over-expressed genes are more likely to form processed pseudogenes [43]. In our analysis, we find that several differentially-expressed pseudogenes are derived from highly-expressed genes: MTATP8P2, MTND4P15, RP1-89D4.1, RP11-380G5.3, AC010733.5. It is likely that these pseudogenes are observed as differentially expressed largely due to fluctuations in the expression of their parent genes.

To investigate the putative function of differentially-expressed pseudogenes that are not derived from over-expressed protein-coding genes, we identified human proteins (Table 2; BLASTX hits against GENCODE using a cutoff E-value of $10^{-4}$) and transcripts/splice variants (Table 3; BLASTN hits against the Ensembl cDNA database using a cutoff E-value of $10^{-4}$ and sequence identity of > 90%) that are similar to these pseudogene transcripts or their translated products.

Several pseudogenes have their parent genes as the best (and sometimes the only) BLASTX or BLASTN hit (Table 2 and Table 3). A pseudogene may have a regulatory function if its expression correlates (positively or negatively) with that of its parent or other similar genes.

**Table 2:** BLASTX hits of differentially-expressed pseudogenes. Only hits to known protein-coding genes with E-value < $10^{-4}$ are shown. “fc” denotes fold change in gene expression; “Mock” represents gene expression in uninfected T-cells; and “HIV” denotes gene expression in HIV-1-infected T-cells 7 days post-infection.

Pseudogene	Chromosome	$\log_{2}$(fc)	BLASTX Hits	Chromosome	Mock	HIV	$\log_{2}$(fc)
**A. Over-expressed**							
DUTP1	3	3.869	DUT	15	181.046	128.614	−0.812
UBE2FP1	3	2.595	UBE2F	2	17.448	20.262	0.216
MTATP8P2	2	2.59	MT-ATP8	MT	1.3e+05	1.5e+05	0.144
MTND4P15	9	2.535	MT-ND4	MT	3.8e+04	3.6e+04	−0.07
RP1-89D4.1	11	2.782	RPS24	10	6416.6	5334.27	−0.267
RP11-720N19.1	17	2.722	MSANTD3	9	9.694	9.455	−0.036
RP11-170L3.6	16	2.595	IGHV4-31	14	0.806	0	-inf
			IGHV4-39	14	-	-	-
RP11-265N6.3	15	2.536	MYL12B	18	100.483	126.451	0.332
			MYL12A	18	255.43	271.675	0.089
**B. Under-expressed**							
ZNF137P	19	−3.092	ZNF816	19	4.178	3.376	−0.307
			ZNF813	19	1.846	0.919	−1.007
			ZNF845	19	4.772	4.083	−0.225
			ZNF83	19	12.51	8.327	−0.587
			FKSG61	14	6.17	6.243	0.017
SCML2P2	16	−3.003	SCMH1	1	6.914	5.049	−0.453
ANTXRLP1	10	−2.865	ANTXRL	10	0.019	0	-inf
HNRNPA3P6	3	−2.812	HNRNPA3	2	330.731	185.908	−0.831
			HNRNPA1	12	1293.29	905.014	−0.515
KLHL2P1	4	−2.611	TMEM135	11	17.787	15.472	−0.201
			KLHL2	4	9.699	11.424	0.236
			MYB	6	87.15	78.048	−0.159
			PKN1	19	25.755	27.804	0.11
CTD-2008A1.2	15	−2.58	SORD	15	25.16	12.187	−1.046
RP11-380G5.3	10	−3.463	RPL11	1	2653.49	2219.2	−0.258
RP11-114F3.5	12	−3.034	HKR1	19	17.206	12.676	−0.441
			CRLF2	X	0.149	1.557	3.387
			TSEN2	3	10.77	6.858	−0.651
			SEPSECS	4	4.842	2.855	−0.762
			MRPS25	3	31.181	28.143	−0.148
			CLTA	9	24.205	34.036	0.492
RP1-224A6.8	1	−2.808	MPHOSPH6	16	22.657	22.301	−0.023
AC010733.5	2	−2.731	RPS12	6	7413.97	8991.73	0.278
RP11-411B10.4	18	−2.632	VN1R4	19	-	-	-
			VN1R2	19	-	-	-
			VN1R1	19	4.273	1.546	−1.466
			LEPRE1	1	6.65	8.324	0.324
RP11-490K7.4	1	−2.613	GTF2A2	15	108.131	143.993	0.413
			STAP2	19	19.65	20.589	0.067
			STARD10	11	49.338	47.267	−0.062
			ADAM10	15	37.318	21.645	−0.786
RP11-471L13.3	17	−2.608	DYNLT1	6	204.754	140.976	−0.539
			DYNLT3	X	16.799	20.654	0.298

**Table 3:** BLASTN hits (transcripts/splice variants) for differentially-expressed pseudogenes. Only hits to known protein-coding genes with E-value < $10^{-4}$ and sequence similarity >90% are shown. “fc” denotes fold change in gene expression; “Mock” represents gene expression in uninfected T-cells; and “HIV” denotes gene expression in HIV-1-infected T-cells 7 days post-infection.

Pseudogene	Chromosome	$\log_{2}$(fc)	BLASTN Hits	Chromosome	Mock	HIV	$\log_{2}$–(fc)
**A. Over-expressed**							
DUTP1	3	3.869	-	-	-	-	-
UBE2FP1	3	2.595	-	-	-	-	-
MTATP8P2	2	2.59	MT-ATP8	MT	1.3e+05	1.5e+05	0.144
			MT-ATP6	MT	133,475	147,477	0.144
MTND4P15	9	2.535	-	-	-	-	-
RP1-89D4.1	11	2.782	RPS24	10	6416.6	5334.27	−0.267
RP11-720N19.1	17	2.722	MSANTD3	9	9.694	9.455	−0.036
RP11-170L3.6	16	2.595	IGHVII-15-1	14	0	0	0
			IGHV4-34	14	0	0	0
RP11-265N6.3	15	2.536	MYL12B	18	100.483	126.451	0.332
**B. Under-expressed**													
ZNF137P	19	−3.092	PIGL	17	39.2801	37.686	−0.06
			TBC1D9B	5	24.227	48.892	1.013
			C2CD3	11	5.705	6.285	0.14
			HLA-DQA1	6	47.241	35.167	−0.426
			ORC6	16	14.991	19.302	0.365
SCML2P2	16	−3.003	-	-	-	-	-
ANTXRLP1	10	−2.865	ANTXRL	10	0.019	0	-inf
			SOD2	6	60.695	112.151	0.886
HNRNPA3P6	3	−2.812	HNRNPA3	2	330.731	185.908	−0.831
KLHL2P1	4	−2.611	KLHL2	4	9.699	11.424	0.236
			FOXK1	7	4.528	6.016	0.41
			DNAJC21	5	25.755	17.957	−0.52
			BIRC5	17	35.427	21.565	−0.716
			TRIM59	3	70.348	43.319	−0.699
CTD-2008A1.2	15	−2.58	SORD	15	25.16	12.187	−1.046
RP11-380G5.3	10	−3.463	DOK1	2	5.483	11.781	1.104
RP11-114F3.5	12	−3.034	IDS	X	2.891	3.413	0.239
			AS3MT	10	20.487	16.317	−0.328
			THAP6	4	7.924	4.931	−0.684
			ZYG11B	1	4.275	2.514	−0.766
			ABCC10	6	8.517	6.681	−0.35
RP1-224A6.8	1	−2.808	MPHOSPH6	16	22.657	22.301	−0.023
AC010733.5	2	−2.731	RPS12	6	7413.97	8991.73	0.278
RP11-411B10.4	18	−2.632	ADC	1	0.768	1.148	0.58
			SLC2A5	1	0.498	0.361	−0.464
			SETD2	3	12.064	9.976	−0.274
RP11-490K7.4	1	−2.613	GTF2A2	15	108.131	143.993	0.413
RP11-471L13.3	17	−2.608	DYNLT1	6	204.754	140.976	−0.539

### 3.2. Pseudogenes with Antagonistic Expression to the Parent Genes

If pseudogene transcripts are acting as molecular sponges for mRNA stability factors and miRNA, then their expression will negatively correlate with that of the parent gene.

We find several pseudogene:gene pairs where pseudogene expression is antagonistic to their BLASTX/BLASTN hits (Table 3 and Table 4). The GO terms for the TBC1D9B protein product suggest its function as a regulator of Rab-GTPase activity. Another GTPase (Rho-GTPase) is required for HIV-1 replication [44,45], but the role of Rab-GTPases in the HIV-1 life cycle is not clear. However, it has been shown that Rab GTPase-activating proteins bind Hepatitis C virus protein NS5A to mediate viral replication [46]. The average gene expression change from nine comparisons at 12 h and 6 h post-infection also show upregulation of TBC1D9B and a >2-fold increase in gene expression seven days post-infection (Table 4). Thus, the interaction between ZNF137P and TBC1D9B transcripts may be implicated in HIV-1 replication.

**Table 4:** Differentially-expressed pseudogenes in early and late HIV-1 infection. $\log_{2}$ (fold change) of only those pseudogene:gene pairs are shown where the parent gene shows a >1.5-fold change in expression due to HIV-1 infection, *i.e.*, $\log_{2}$ (fold change) either >0.585 (upregulated) or <−0.585 (downregulated). The time points (12 h, 24 h or 7 days post-infection) where the genes show a >1.5-fold change in expression are highlighted in bold. The genes that are upregulated or downregulated in all of the replicate observations (nine observations for 12 h and six observations for 24 h time-points) are identified by *. “on” genes are those that show detectable expression in HIV-1-infected cells only, and “off” genes show detectable expression in uninfected cells only. The $\log_{2}$ (fold change) at the 12-h and 24-h time points is the average $\log_{2}$ (fold change) over replicate observations. Supplementary Table S2 lists $\log_{2}$ (fold change) in each replicate. ANTXRL did not have detectable gene expression in any of the nine observations at 12 h post-infection.

Pseudogene	12 h	24 h	7 d	Parent Gene	12 h	24 h	7 d
DUTP1	**−0.908**	on	**3.869**	DUT	0.245	−0.527 *	**−0.812**
RP11-265N6.3	**1.082**	on	**2.536**	MYL12A	0.619 *	−0.624 *	0.089
ZNF137P	−0.031	**−1.007**	**−3.092**	TBC1D9B	0.229	0.18 *	**1.013**
				C2CD3	**−0.6**	−0.21 *	0.14
				ZNF813	−0.212	−0.298 *	**−1.007**
				ZNF83	0.062	0.62 *	**−0.587**
ANTXRLP1	off	**−3.75**	**−2.865**	ANTXRL	-	**−1.086**	off
HNRNPA3P6	0.406	**−3.517**	**−2.812**	HNRNPA3	−0.231	−0.962 *	**−0.831**
				HNRNPA1	−0.14	−0.903 *	−0.515
KLHL2P1	on	0.017	**−2.611**	KLHL2	−0.043	**0.79**	0.236
				FOXK1	−0.68 *	−1.075 *	0.41
				BIRC5	−0.238	−1.163 *	**−0.716**
				TRIM59	−0.1	−0.22	**−0.699**
				MYB	−0.694 *	−1.915 *	−0.159
CTD-2008A1.2	0.131	−0.319	**−2.58**	SORD	0.055	−1.928 *	**−1.046**
RP11-380G5.3	−0.083	0.285	**−3.463**	DOK1	0.494	−0.252 *	**1.104**
RP11-114F3.5	−0.438	−0.0628	**−3.034**	CRLF2	**−1.08**	on *	**3.387**
				TSEN2	−0.423 *	−1.647 *	**−0.651**
				SEPSECS	−0.003	−0.213 *	**−0.762**
				CLTA	−0.05	−1.255 *	0.492
				THAP6	0.474 *	0.806 *	**−0.684**
				ZYG11B	−0.099	−0.093	**−0.766**
				ABCC10	−0.594 *	0.419 *	−0.35
RP1-224A6.8	0.096	on	**−2.808**	MPHOSPH6	0.123	−0.618 *	−0.023
RP11-411B10.4	0.02	−0.53	**−2.632**	ADC	0.24	3.235 *	0.58
				SLC2A5	**−1.698**	1.908 *	−0.464
				VN1R1	0.296 *	−0.425 *	**−1.466**
RP11-490K7.4	0.346	0.069	**−2.613**	GTF2A2	0.031	−0.83*	0.413
				ADAM10	−0.115	−0.12	**−0.786**
				STAP2	−0.358	**0.704**	0.067
RP11-471L13.3	on	**0.839**	**−2.608**	DYNLT1	0.323 *	0.626 *	−0.539
				DYNLT3	−0.099	−0.67 *	0.298

It is known that HIV’s Tat protein downregulates SOD2 transcripts in mitochondria, leading to mitochondria-mediated apoptosis in infected cells [47], making the pair ANTXRLP1:SOD2 of interest (Table 3).

RP11-411B10.4’s parent gene ADC is upregulated in HIV-1 infection (more than an eight-fold increase in gene expression at the 24-h time point; Table 4). ADC encodes arginine decarboxylase, an enzyme that metabolizes L-arginine to agmatine. This is interesting, because increased metabolism of L-arginine impairs lymphocyte response to antigens [48]. Thus, even though ADC expression is not linked to HIV-1 infection, upregulation of arginine decarboxylase appears to be an effective viral strategy to evade host immune response.

### 3.3. Pseudogenes with Synergistic Expression to the Parent Genes

Sometimes, pseudogene transcripts compete with the parent gene transcript for silencing non-coding RNAs, as has been shown for tumor suppressor gene PTEN and its pseudogene PTENP1 [20]. Pseudogene and parent gene (or other genes with sequence similarity to the pseudogene) expression are positively correlated under this model of pseudogene:gene interaction. In HIV-1 infection, we find the following pseudogene:gene pairs with a positively correlated decrease in expression: ZNF137P:ZNF813, ANTXRLP1:ANTXRL, HNRNPA3P6:HNRNPA3/HNRNPA1, CTD-2008A1.2:SORD, KLHL2P1:BIRC5, KLHL2P1:TRIM59, KLHL2P1:MYB, RP11-114F3.5:TSEN2, RP11-114F3.5:SEPSECS, RP11-114F3.5:ZYG11B and RP11-490K7.4:ADAM10.

Of these, only a few parent genes code for proteins that are known to have a role in viral infection, such as HNRNPA3/HNRNPA1. The heterogeneous nuclear ribonucleoproteins (hn-RNPs) bind to pre-mRNA and are involved in presenting the mRNA to the splicing machinery and transporting mRNA to the cytoplasm. Downregulating hn-RNP A proteins promotes viral production due to efficient alternative splicing of viral mRNA transcripts and their trafficking [49]. Further, upregulation of hn-RNP A1 results in a 100-fold decline in virus production [50]. We find that pseudogene HNRNPA3P6, its parent gene HNRNPA3 and a close paralog of the parent gene HNRNPA1 are under-expressed in HIV-1 infection (Table 2 and Table 3). It is thus possible that the virus exploits the regulatory influence of pseudogene HNRNPA3P6 on HNRNPA3 and HNRNPA1 to downregulate hn-RNP proteins and promote virus production.

Several functional genes similar to pseudogene KLHL2P1 are downregulated in HIV-1 infection (Table 4). BIRC5 inhibits apoptosis, and its downregulation in both early and late HIV-1 infection could contribute to HIV-associated depletion of T-cells [51]. While the TRIM family of proteins is important in innate immune responses [52], we find TRIM59 to be downregulated in HIV-1 infection (Table 4). MYB encodes a transcriptional activator protein that binds to the HIV-1 long terminal repeat (LTR) and activates viral transcription [53]. Consistent downregulation of MYB expression could thus be the host response to mitigate HIV-1 transcription.

Pseudogene RP11-490K7.4’s functional relative ADAM10 encodes a cell surface protein that has both adhesion and protease domains and has been shown to facilitate nuclear trafficking of HIV-1 [54]. Another protein coding gene similar to this pseudogene, GTF2A2, is found downregulated in uninfected T-cells in response to exposure to the HIV-1 gp120/V3 epitope [55], similar to our observation at 24 h post-infection.

### 3.4. Pseudogenes and Their Parent Genes with Modulating Gene Expression in Early and Late HIV-1 Infection

As the expression of pseudogene DUTP1 goes from a >1.5-fold decrease at 12 h post-infection to a >8-fold increase at seven days post-infection, its parent gene expression is modulated as well from over-expressed to under-expressed (Table 4). DUT codes for a protein that hydrolyzes the dUTP to dUMP reaction, and its downregulation will increase cellular dUTP levels. Heavy uricilation of HIV reverse transcripts is common and promotes chromosomal integration of the viral genome [56]. Thus, over-expression of DUTP1 to downregulate DUT expression may be the cellular response to inhibit the integration of uricilated viral transcripts into the host genome.

Pseudogene RP11-265N6.3 is consistently upregulated in HIV-1 infection with consistent expression at 12 h and 24 h, and a >4-fold increase in expression at seven days post-infection. However, its parent gene MYL12A shows modulating gene expression at 12 h and 24 h post-infection. MYL12A is involved in the regulation of the actin cytoskeleton and is found to be upregulated in HIV/HCV co-infection [57]. HIV-1 proteins are known to reorganize the actin cytoskeleton to optimize virion production [58].

While pseudogene ZNF137P is increasingly downregulated as the infection progresses, its parent gene ZNF83, an anti-inflammatory gene [59], is over-expressed by a factor of 1.5 in all six datasets at 24 h post-infection, but is under-expressed seven days post-infection (Table 4). In contrast, FOXK1 (parent gene of KLHL2P1) is downregulated only in early viral infection (a >1.5-fold decrease in expression and downregulation in all nine datasets at 12 h and six datasets at the 24-h time point; Table 4). FOXK1 inhibited interferon production in Sendai virus infection, a single-stranded RNA virus [60], and its downregulation in early HIV-1 infection likely contributes to triggering the immune response.

In the RP11-114F3.5:CRLF2 pair, the CRLF2 gene shows a >2-fold downregulation 12 h post-infection, followed by an increase in gene expression at 24 h (as reflected by the “on” signal in all six transcriptomic comparisons at 24 h post-infection), leading to a >8-fold increase in expression in infected cells seven days post-infection (Table 4). Thus, it appears that the CRLF2 gene expression went from downregulated in early infection to significantly upregulated in the late HIV-1 infection. CRLF2 encodes a receptor for cytokine TSLP, whose over-production in HIV-1 infected mucosal epithelial cells amplifies HIV infection in activated CD4+ T-cells [61]. It is thus important to understand how CRLF2 expression is regulated in T-cells and if pseudogene RP11-114F3.5 has any influence on its post-transcriptional regulation.

ABCC10, a functional protein similar to pseudogene RP11-114F3.5, codes for the multidrug resistance-associated protein 7 (MRP7) that transports molecules, such as xenobiotics, across intra- and extra-cellular membranes [62]. HIV-Tat is known to upregulate the expression of another transporter from the same family (MRP1), a cell-surface protein of the cells of the blood brain barrier that remove anti-viral drugs from the central nervous system [63]. It is thus likely that modulating gene expression of ABCC10 (downregulated in all nine datasets at 12 h and upregulated in all six datasets at 24 h post-infection) might be interfering with the transport of molecules across HIV-1-infected T-cells. Interestingly, another transporter encoding gene (SLC2A5) shows modulating gene expression, as well (a >3-fold decrease in expression at 12 h and a >3-fold increase in expression at the 24-h time point; Table 4).

Pseudogene RP11-471L13.3 is over-expressed by a factor of >1.5 at 24 h and shows a >4-fold decrease in expression seven days post-infection. Its putative parents genes (DYNLT1 and DYNLT3) are dynein motor proteins that carry cargo, such as proteins, across the cellular microtubules; dynein proteins are thought to associate with viral proteins during infections [64].

Other pseudogene:gene pairs with modulating gene expression in early and late HIV-1 infection, but with unknown roles in viral infection, are RP11-380G5.3:DOK1 and RP11-114F3.5:THAP6.

## 4. Discussion

Although Vanin characterizes pseudogenes as “related but defective” [22], this sentiment has seen a shift in recent years. With the accumulating evidence of functional consequences of pseudogene transcription, the term “pseudogene” should be revisited [1]. A pseudogene capable of exerting regulatory control on other genes via its RNA intermediates is functionally active even if it does not code for a protein. Although most pseudogenes are thought to evolve neutrally [3,65], some transcribed pseudogenes exhibit patterns of non-neutral evolution [66]. The contribution of pseudogenes to the non-coding small-RNA pool that regulates the expression of a wide range of genes provides a possible explanation for their evolutionary maintenance. About a fifth of human pseudogenes are transcribed [3], and thus might be candidates for purifying selection if they participate in gene expression regulation. For a retrovirus with a pre-packaged reverse transcriptase and integrase, this presents an opportunity to exploit a layer of gene expression regulation for its own benefit. Here, we present evidence that suggests the involvement of pseudogenes in the regulation of cellular gene expression in the early stages of a retroviral infection.

Our computational pipeline for detecting differentially-expressed pseudogenes relies on software that is widely used and accepted by the scientific community. Gene expression is normalized by gene length to account for the fact that longer genes end up producing more sequencing reads than shorter genes. Although it is desirable to have replicates in the sequencing experiment design, replicate data are usually generated by pooling the replicate samples together in a single sequencing run, reducing the data generated per sample. While we analyzed multiple replicate transcriptomes at 12 h and 24 h post-infection (which likely increased specificity) the single transcriptome at seven days post-infection time point had four times the sequencing data (≈80 million reads compared to the ≈20 million reads at the 12-h and 24-h time points). This high sequencing depth combined with high quality reads and a mean fragment size of >350 (150 × 2 paired-end sequencing) for both uninfected and infected samples improves sensitivity in detecting differentially-expressed genes and adds confidence to our predictions. Both increasing sequencing depth and biological replication have been shown to increase the power of detecting differentially-expressed genes [67]. Strongly upregulated protein-coding genes in our data (the seven-day time point) are implicated in HIV-1 infection, lending further confidence to our analysis (Supplementary Table S1).

While our methodology cannot find novel pseudogenes that might be created in an infected cell due to viral reverse transcriptase and integrase activity, we find a small number of known pseudogenes that are differentially expressed in HIV-1-infected cells. While the data from the 12-h and 24-h time points comes from a T lymphocyte cell-line that is different from the T-cell-line at the seven-day time point, our candidate pseudogene:gene interactions show consistent differential expression across the three time points, suggesting that these host responses are not cell-line specific. We further identify protein-coding genes (parent genes or genes that are BLASTX/BLASTN hits to the pseudogenes) whose expression is correlated with that of the differentially-expressed pseudogenes. One third of these parent genes are implicated in HIV-1 infection, suggesting pseudogene-mediated post-transcriptional regulation of gene expression in the infected cells. It should be noted that the study that experimentally confirmed the effect of pseudogene PTENP1 transcription on the mRNA levels of tumor suppressor gene parent gene PTEN showed that the change in PTEN mRNA expression was less than two-fold, yet the pseudogene PTENP1 is “selectively lost in human cancer” [20]. Thus, we restricted our focus to those parent genes that show a >1.5-fold change in gene expression at 12 h, or 24 h, or seven days post-infection (Table 4). It is possible that these parent genes are differentially expressed in HIV-1 infection due to other factors in the complex gene-regulatory machinery of the cell, but the significant change in expression in their pseudogenes suggests pseudogene-mediated gene expression regulation. Experimental validation of regulatory interactions between the candidate pseudogene:gene pairs is needed to confirm if viruses exploit this newly-discovered layer of post-transcriptional regulation of gene expression.

## 5. Conclusions

In this study, we find that instead of being silent spectators, pseudogenes are differentially expressed in HIV-1 infection. While non-coding RNAs are now considered integral to the host response to viral infections, our findings indicate for the first time that RNAs derived from pseudogene transcripts are a part of the cellular non-coding RNA pool, and may regulate expression of genes implicated in viral infections. Functional relevance of pseudogene transcription has been experimentally verified in cancer but remains to be validated in viral infections. We anticipate that pseudogene transcription will be of significance in other cellular processes and in host responses to the environment as well.

## Supplementary Files

Supplementary File 1
