# Supplementary material for: Differentially-Expressed Pseudogenes in HIV-1 Infection
Source: Viruses. 2015 Sep 29;7(10):5191–205. doi: 10.3390/v7102869 (PMC4632377; doi:10.3390/v7102869)
Supplement: Supplementary File 1 [file viruses-07-02869-s001.pdf]

Supplementary Information

Differentially Expressed Pseudogenes in HIV-1 Infection

Aditi Gupta [1,2,*], C. Titus Brown [1,2,3], Yong-Hui Zheng [1,2] and Christoph Adami [1,2,4]

Received: 18 July 2015 / Accepted: 18 September 2015 / Published: 29 September 2015
Academic Editor: Andrew Mehle

[1] Department of Microbiology and Molecular Genetics, Michigan State University, 567 Wilson Road, East Lansing, MI 48824, USA; ctbrown@ucdavis.edu (C.T.B.); zhengyo@msu.edu (Y.-H.Z.); adami@msu.edu (C.A.)
[2] BEACON Center for the Study of Evolution in Action, Michigan State University, 567 Wilson Road, East Lansing, MI 48824, USA
[3] Department of Computer Science and Engineering, Michigan State University, 428 S. Shaw Lane, East Lansing, MI 48824, USA
[4] Department of Physics and Astronomy, Michigan State University, 567 Wilson Road, East Lansing, MI 48824, USA
* Correspondence: agupta@msu.edu; Tel.: +1-517-355-8733, Fax: +1-517-353-8957

Supplementary Material

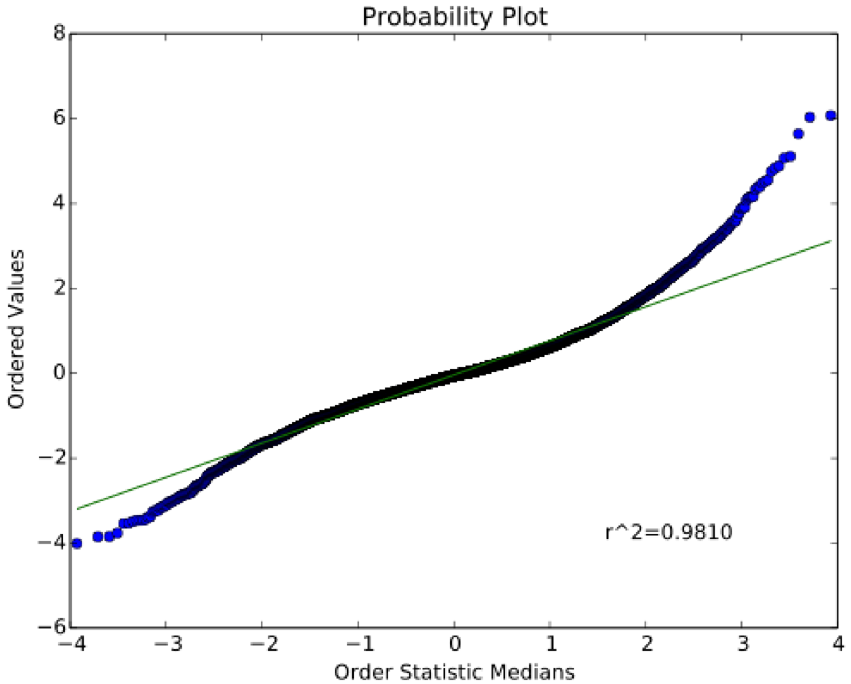

Figure S1. QQ plot of log_2 fold-change in gene-expression shows a strong fit to normal distribution. The plot was generated using the stats.probplot() function of SciPy.

Table S1. Protein-coding genes strongly over-expressed in HIV-1 infection. Top 15 protein-coding genes up-regulated in HIV-1 infection (ranked by $\log_2$ fold-change in gene-expression). NI indicates gene-expression in uninfected H9 T-cells and ND denotes gene-expression in HIV-1 infected T-cells.

Gene	Gene Name	NI	ND	Log_2 (Fold-Change)	Note
EMP1	Epithelial Membrane Protein 1	0.564	37.986	6.074	EMP1 is a tight junction protein of blood-brain barrier [1], and tight-junctions are disrupted in HIV-1 infection [2–4].
VGF	Nerve Growth Factor Inducible	0.233	15.279	6.036	Linked to cognitive impairment [5] and macrophage survival [6] in HIV infection, recombinant version used to treat sensory neuropathy in HIV infection [7].
CSF2	Colony Stimulating Factor 2	0.638	31.921	5.645	Encodes a cytokine that regulates production and function of granulocytes and macrophages, linked to HIV infection [8].
IFIT2	Interferon-induced Protein with Tetratricopeptide Repeats 2	0.986	29.184	4.887	As one of ISGs (Interferon Stimulated Genes), this gene has antiviral activity [9,10] and is linked to HIV-1 infection [11,12].
ARC	Activity-Regulated Cytoskeleton-associated Protein	0.302	8.61	4.834	HIV-1 uses cytoskeletal components to traffic viral particles in host-cell cytoplasm [13] and to sensitize T-cells for apoptosis [14].
ZNF365	Zinc Finger Protein 365	0.104	2.789	4.752	Linked to HIV-1 genome integration process [15].
PHLDA1	Pleckstrin Homology-Like Domain, family A, member 1	2.124	50.01	4.557	Activated by insulin-like growth-factor 1 (has anti-apoptotic effects), linked to HIV infection [16].
RRAD/RAD1	Ras-Related Associated with Diabetes	0.369	8.437	4.516	Linked to DNA damage response activated by HIV-1 protein vpr [17,18].
OASL	2'-5'-Oligoadenylate Synthetase-Like	1.281	28.681	4.484	Interferon-induced RNA response gene, differentially expressed in HIV-1 infection [19,20].
CXCL11	Chemokine (C-X-C motif) Ligand 11	0.847	17.742	4.389	Involved in leukocyte trafficking, recruits CD4+ T-cells to HIV-1 infected cells [21].
RASL11A	RAS-Like, family 11, member A	0.172	3.588	4.383	Belongs to small GTPase family, highly similar to RAS. HIV-1 has high Ras-responsiveness [22,23]. Ras pathway synergestically activates NFAT (nuclear factor of activated T cells) with HIV-1 protein Nef [24].

Table S1. *Cont.*

Gene	Gene Name	NI	ND	Log_2 (Fold-Change)	Note
GJB2	Gap Junction Protein, beta 2	0.253	4.483	4.15	Gap junctions are critical in spreading toxicity mediated by HIV-infected astrocytes, leading to neurological dysfunction [25]. They are also important in cell-cell communication during HIV infection [26].
GJB6	Gap Junction Protein, beta 6	0.13	2.27	4.123	Same as GJB2, important in blood-tissue barriers [27].
MMP7	Matrix Metallopeptidase 7	0.826	13.917	4.075	Limits HIV-induced neurotoxicity [28]. Are over-expressed in HIV infection [29,30].
IL2	Interleukin-2	4.955	74.738	3.915	Cytokine important for T and B cell proliferation. Linked to immune response to HIV-1 infection [31].

Table S2. Log_2 (fold-change) in gene expression of pseudogenes and their parent genes at 12 h and 24 h post infection. The transcriptomics data was accessed from the GEO database (GEO ID: GSE53993). There are 9 comparisons for 12 h time-point (3 mock transcriptomes compared with 3 HIV-1 infected transcriptomes) and 6 comparisons for 24 h time-point (2 mock transcriptomes compared with 3 HIV-1 infected transcriptomes). Undetectable gene-expression in both mock and HIV-1 infected cell is denoted as "NA"; detectable gene-expression in HIV-1 infected cells is termed as the gene being turned "on" due to infection; and detectable gene-expression in mock dataset only is termed as gene being "off" in HIV-1 infection. The reference human transcriptome has multiple entries for certain genes (for example HLA-DQA1), and as a result, these genes have >9 data points at 12 h and >6 data points at 24 h time-point: these genes are ignored and not considered in further analyses.

Pseudogene/Parent Gene	Log_2 (Fold-Change) at 12 h Post-Infection (9 Datasets)	Log_2 (Fold-Change) at 24 h Post-Infection (6 Datasets)
RP11-720N19.1	"NA", "off", "NA", "on", −0.187, "on", "NA", "off", "NA"	"on", "on", "on", "on", "on", "on"
RPS12	0.197, 0.463, −0.094, 0.434, 0.668, 0.105, 0.184, 0.411, −0.15	−0.306, −0.663, 0.009, −0.297, −0.399, −0.765
CRLF2	"NA", "off", "off", "NA", "off", "off", "on", −1.104, −1.055	"on", "on", "on", "on", "on", "on"
FOXK1	−0.969, −0.667, −0.915, −0.422, −0.152, −0.406, −0.954, −0.693, −0.942	−1.013, −0.977, −0.856, −0.772, −1.428, −1.406
TMEM135	0.171, 0.021, 0.032, 0.323, 0.143, 0.145, −0.127, −0.311, −0.31	0.018, 0.237, 0.069, 0.339, −0.39, −0.178
RP1-89D4.1	1.15, 1.472, 1.556, 2.122, 2.41, 2.486, −1.154, −0.871, −0.793	−2.097, −0.312, −1.795, 0.045, −0.604, 1.177

Table S2. *Cont.*

Pseudogene/Parent Gene	Log_2 (Fold-Change) at 12 h Post-Infection (9 Datasets)	Log_2 (Fold-Change) at 24 h Post-Infection (6 Datasets)
BIRC5	0.082, −0.135, −0.04, −0.121, −0.368, −0.281, −0.285, −0.54, −0.451	−1.43, −1.371, −1.022, −0.911, −1.149, −1.097
FKSG61	−0.427, −0.562, 0.224, −0.921, −1.092, −0.31, −0.587, −0.756, 0.018	−0.584, 0.242, −0.411, 0.466, 0.159, 0.981
IDS	0.946, −0.514, −0.034, “on”, 0.298, −0.096, 0.047, −2.791, −0.966, “on”, −0.64, −2.411, −0.078, −0.806, −1.095, “on”, −0.771, −0.432	0.074, −1.176, 0.125, −0.745, −0.381, 0.574, −0.276, 1.059, 0.927, 0.121, 0.974, 0.548
DNAJC21	−1.212, −1.305, −0.218, −0.536, −0.661, 0.418, −0.453, −0.577, 0.497	−0.026, −0.17, −0.073, −0.161, 0.272, 0.126
ADAM10	−0.349, −0.22, 0.242, −0.102, −0.002, 0.449, −0.565, −0.468, −0.02	−0.332, −0.224, −0.032, 0.132, −0.185, −0.079
HNRNPA3P6	0.598, 1.174, 0.838, 0.469, 1.017, 0.67, −0.615, −0.079, −0.418	“off”, “off”, −3.511, −3.523, “off”, “off”
RP11-490K7.4	1.402, 0.661, 0.296, 0.285, −0.488, −0.861, 1.249, 0.468, 0.101	−1.533, 0.636, −0.526, 1.697, “off”, “off”
GTF2A2	0.082, 0.453, −0.082, 0.16, 0.499, −0.045, −0.301, 0.025, −0.508	−1.261, −1.239, −0.861, −0.787, −0.424, −0.408
RP11-265N6.3	“on”, “on”, 1.588, “NA”, “NA”, “off”, “on”, “on”, 0.575	“on”, “on”, “on”, “on”, “NA”, “NA”
ZNF813	−0.262, −0.816, −0.062, 0.191, −0.394, 0.351, −0.162, −0.749, −0.006	−0.171, −0.211, −0.436, −0.426, −0.248, −0.297
RP11-380G5.3	−0.397, −1.085, −0.812, 0.498, −0.221, 0.044, 0.801, 0.082, 0.343	−0.311, 1.179, −0.277, 1.265, −0.815, 0.671
STARD10	0.046, −0.272, −0.144, 0.351, 0.001, 0.122, 0.219, −0.129, −0.014	0.282, −0.152, 0.46, 0.072, 0.453, 0.007
LEPRE1	0.618, 0.436, 0.523, 0.218, 0.004, 0.085, 0.363, 0.145, 0.226	−0.637, −0.968, −0.123, −0.408, −0.061, −0.404
RP1-224A6.8	“off”, “off”, “off”, “off”, “off”, “off”, 0.904, −0.99, 0.375	“on”, “on”, “on”, “on”, “NA”, “NA”
ZNF816	0.487, −0.043, 0.28, −0.001, −0.56, −0.248, −0.035, −0.598, −0.285	0.22, −0.154, 0.331, 0.01, 0.523, 0.144
MTATP8P2	“NA”, “NA”, “NA”, “NA”, “NA”, “NA”, “NA”, “NA”, “NA”	“on”, “on”, “NA”, “NA”, “NA”, “NA”
ANTXRL	“NA”, “NA”, “NA”, “NA”, “NA”, “NA”, “NA”, “NA”, “NA”	“off”, “NA”, “off”, “NA”, −1.086, “on”

Table S2. *Cont.*

Pseudogene/Parent Gene	Log2 (Fold-Change) at 12 h Post-Infection (9 Datasets)	Log2 (Fold-Change) at 24 h Post-Infection (6 Datasets)
MYL12A	0.569, 0.892, 0.416, 0.556, 0.848, 0.363, 0.611, 0.9, 0.413	−0.685, −0.962, −0.356, −0.577, −0.444, −0.722
TRIM59	−0.204, −0.428, 0.035, 0.13, −0.127, 0.33, −0.191, −0.448, 0.004	−0.51, −0.598, −0.253, −0.283, 0.206, 0.119
STAP2	0.038, 0.541, 0.602, −1.238, −0.77, −0.709, −0.888, −0.428, −0.367	1.997, 1.112, 0.642, −0.196, 0.784, −0.113
DYNLT1	0.445, 0.571, 0.255, 0.312, 0.404, 0.082, 0.328, 0.418, 0.095	0.505, 0.407, 0.774, 0.727, 0.723, 0.618
AC010733.5	0.211, −1.579, −1.054, 1.584, −0.24, 0.282, 1.438, −0.384, 0.13	−0.304, 0.435, 0.84, 1.631, −1.353, −0.62
DYNLT3	−0.076, −0.415, −0.144, 0.384, 0.011, 0.274, −0.144, −0.527, −0.255	−0.643, −0.886, −0.708, −0.892, −0.324, −0.567
AS3MT	0.164, −0.406, 0.146, 0.445, −0.156, 0.386, 0.076, −0.529, 0.014	−0.021, 0.081, 0.436, 0.593, 0.552, 0.652
VN1R2	1.482, 0.698, 0.535, 0.335, −0.483, −0.651, 1.317, 0.498, 0.326	1.758, 1.018, “off”, “off”, “off”, “off”
PKN1	0.187, −0.325, 0.157, 0.184, −0.36, 0.118, 0.515, −0.023, 0.44	−0.736, −1.006, −1.115, −1.341, −0.417, −0.703
VN1R1	0.268, 0.244, 0.138, 0.604, 0.549, 0.436, 0.221, 0.162, 0.046	−0.292, −0.446, −0.32, −0.418, −0.46, −0.616
ORC6	−0.099, −0.447, 0.201, −0.147, −0.525, 0.114, −0.205, −0.589, 0.051	−0.843, −0.51, −0.614, −0.231, −0.239, 0.085
TSEN2	−0.558, −0.595, −0.638, −0.062, −0.131, −0.18, −0.482, −0.555, −0.608	−1.515, −1.961, −1.114, −1.505, −1.668, −2.119
VN1R4	“off”, “NA”, “NA”, “off”, “NA”, “NA”, “off”, “NA”, “NA”	“on”, −0.401, “NA”, “off”, “on”, 0.259
MTND4P15	1.463, −0.442, 1.615, −0.607, −2.553, −0.495, 2.165, 0.221, 2.272	“on”, “on”, “on”, “on”, “on”, “on”
RP11-170L3.6	“NA”, “NA”, “NA”, “NA”, “NA”, “NA”, “NA”, “NA”, “NA”	“NA”, “NA”, “NA”, “NA”, “NA”, “NA”
IGHV4-34	1.116, “NA”, “on”, “NA”, −0.109, “NA”, 0.196, “NA”, “on”, “NA”, −1.067, “NA”, −0.088, “NA”, “on”, “NA”, −1.355, “NA”	“off”, “NA”, “NA”, “NA”, −1.65, “NA”, “on”, “NA”, −1.501, “NA”, “on”, “NA”
MYB	−1.034, −1.169, −0.643, −0.549, −0.715, −0.197, −0.708, −0.875, −0.359	−2.119, −1.94, −1.976, −1.744, −1.941, −1.767

Table S2. *Cont.*

Pseudogene/Parent Gene	Log2 (Fold-Change) at 12 h Post-Infection (9 Datasets)	Log2 (Fold-Change) at 24 h Post-Infection (6 Datasets)
ANTXRLP1	“NA”, “NA”, “NA”, “NA”, “off”, “NA”, “NA”, “NA”, “NA”, “NA”, “off”, “NA”, “NA”, “NA”, “NA”, “NA”, “off”, “NA”	−3.75, “NA”, “on”, “NA”, “off”, “NA”, “NA”, “NA”, “off”, “NA”, “NA”, “NA”
IGHV4-31	“on”, “NA”, “on”, “NA”, 0.449, “off”, “on”, “NA”, “on”, “NA”, 0.498, “off”, “on”, “on”, “on”, “on”, −1.842, −1.454	−2.389, “NA”, −0.184, “NA”, “off”, “NA”, “off”, “NA”, “off”, “NA”, “off”, “NA”
C2CD3	−1.194, −1.232, −0.347, −0.673, −0.744, 0.135, −0.69, −0.765, 0.113	−0.119, −0.075, −0.501, −0.405, −0.099, −0.063
RP11-471L13.3	“NA”, “NA”, “NA”, “NA”, “NA”, “NA”, “on”, “on”, “on”	0.595, “on”, “off”, “NA”, 1.083, “on”
RPS24	0.348, −0.338, 0.352, 0.44, −0.277, 0.4, 0.844, 0.127, 0.804	−0.728, −0.639, 0.254, 0.398, 0.301, 0.387
HNRNPA1	−0.102, −0.245, −0.008, 0.057, −0.117, 0.112, −0.277, −0.456, −0.227	−1.271, −0.935, −1.01, −0.62, −0.957, −0.626
SOD2	0.052, −0.042, −0.054, 0.402, 0.278, 0.256, 0.159, 0.025, 0.009	−0.161, −0.08, −0.019, 0.113, −0.072, 0.001
IGHVII-15-1	“NA”, “NA”, “NA”, “NA”, “NA”, “NA”, “NA”, “NA”, “NA”, “NA”, “NA”, “NA”, “NA”, “NA”, “NA”, “NA”, “NA”, “NA”	“NA”, “NA”, “NA”, “NA”, “NA”, “NA”, “NA”, “NA”, “on”, “NA”, “on”, “NA”
ZNF83	0.486, −0.031, 0.352, 0.329, −0.219, 0.155, 0.072, −0.482, −0.106	0.553, 0.707, 0.303, 0.511, 0.747, 0.897
HNRNPA3	−0.223, −0.736, 0.102, 0.023, −0.517, 0.309, −0.256, −0.81, 0.026	−1.193, −1.055, −1.119, −0.924, −0.807, −0.671
RP11-114F3.5	−0.668, −0.644, −0.129, −0.872, −0.881, −0.373, −0.288, −0.298, 0.207	“off”, “off”, −1.621, −0.057, −0.04, 1.467
PIGL	−0.432, −0.304, −0.762, 0.278, 0.374, −0.09, 0.022, 0.115, −0.354	−0.198, −0.424, −0.036, −0.211, 0.003, −0.231
SORD	−0.091, 0.415, −0.337, 0.141, 0.614, −0.143, −0.093, 0.377, −0.384	−1.997, −2.061, −1.922, −1.935, −1.79, −1.862
ZYG11B	0.064, −0.444, 0.12, 0.265, −0.276, 0.28, −0.121, −0.669, −0.109	−0.403, −0.26, −0.276, −0.076, 0.158, 0.299
CLTA	−0.011, −0.381, 0.038, 0.399, −0.004, 0.407, −0.167, −0.575, −0.16	−1.498, −1.5, −1.069, −1.021, −1.216, −1.227
DUTP1	“NA”, “off”, “off”, “NA”, “off”, “off”, “on”, −0.686, −1.13	“NA”, “NA”, “on”, “on”, “on”, “on”
KLHL2	−0.472, −0.076, 0.382, −0.132, 0.232, 0.685, −0.726, −0.363, 0.086	1.5, −0.142, 1.455, −0.135, 1.855, 0.207

Table S2. *Cont.*

Pseudogene/Parent Gene	Log_2 (Fold-Change) at 12 h Post-Infection (9 Datasets)	Log_2 (Fold-Change) at 24 h Post-Infection (6 Datasets)
RPL11	0.216, 0.089, 0.093, 0.435, 0.277, 0.272, 0.184, 0.024, 0.017	−0.867, −0.792, −0.485, −0.358, −0.436, −0.366
SCMH1	−0.32, −1.129, −0.5, 0.438, −0.407, 0.219, 0.133, −0.716, −0.089	0.065, −0.5, 0.219, −0.297, 0.323, −0.252
SETD2	−0.321, −0.666, 0.145, −0.124, −0.5, 0.303, −0.478, -0.856, −0.055	−0.453, −0.345, −0.302, −0.139, 0.229, 0.335
DUT	−0.17, 0.582, −0.288, 0.455, 1.178, 0.298, −0.134, 0.58, −0.294	−0.139, −0.193, −1.035, −1.037, −0.349, −0.411
RP11-411B10.4	−0.492, 0.454, −0.25, 0.157, 1.069, 0.359, −0.739, 0.165, −0.54	−0.768, −0.133, −0.525, 0.164, −1.274, −0.644
ABCC10	−0.582, −0.387, −0.569, −0.382, −0.22, −0.407, −0.973, −0.818, −1.004	0.42, 0.609, 0.269, 0.506, 0.265, 0.442
KLHL2P1	"on", "on", "on", "NA", "NA", "NA", "NA", "NA", "NA"	"off", "NA", −0.097, "on", 0.131, "on"
SCML2P2	"NA", "NA", "NA", "NA", "NA", "NA", "on", "on", "on"	"NA", "off", "NA", "off", "NA", "off"
MYL12B	0.049, 0.318, −0.046, 0.369, 0.607, 0.236, −0.298, −0.065, −0.434	−0.429, −0.569, −0.361, −0.447, −0.398, −0.545
THAP6	0.59, 0.283, 0.721, 0.659, 0.321, 0.752, 0.397, 0.056, 0.485	0.744, 0.436, 1.132, 0.88, 0.977, 0.668
ADC	1.476, 0.285, 0.207, 0.032, −1.185, −1.276, 1.719, 0.5, 0.401	3.106, 3.929, 3.035, 3.908, 2.308, 3.123
UBE2FP1	−2.078, −1.624, −2.16, "off", "off", "off", −1.097, −0.675, −1.224	"on", 0.288, "on", 0.887, "NA", "off"
SLC2A5	−1.925, −0.76, 0.503, −4.527, −3.396, −2.14, −2.182, −1.054, 0.202	1.428, 2.616, 1.155, 2.395, 1.336, 2.517
MRPS25	−0.555, 0.053, −0.198, 0.457, 1.032, 0.777, −0.189, 0.379, 0.126	−0.822, −0.301, −0.456, 0.117, −1.071, −0.559
ZNF845	−0.113, −0.433, −0.241, 0.124, −0.225, −0.043, −0.078, −0.423, −0.248	−0.495, −0.325, −0.806, −0.585, −0.575, −0.411
UBE2F	0.272, 0.211, 0.293, 0.373, 0.28, 0.355, 0.156, 0.057, 0.133	−0.578, −0.954, −0.277, −0.602, −0.279, −0.663
TBC1D9B	0.142, −0.191, 0.141, 0.501, 0.137, 0.461, 0.428, 0.059, 0.383	0.07, 0.199, 0.092, 0.27, 0.164, 0.283
HKR1	0.377, 0.26, 0.071, 0.305, 0.159, −0.041, 0.25, 0.098, −0.1	−0.172, −0.266, −0.483, −0.528, −0.001, −0.107

Table S2. *Cont.*

Pseudogene/Parent Gene	Log$_2$ (Fold-Change) at 12 h Post-Infection (9 Datasets)	Log$_2$ (Fold-Change) at 24 h Post-Infection (6 Datasets)
MPHOSPH6	0.108, −0.419, 0.126, 0.461, −0.099, 0.439, 0.359, −0.201, 0.335	−1.299, −1.086, −0.651, −0.384, −0.249, −0.039
CTD-2008A1.2	−0.445, −1.329, −0.133, 0.12, −0.799, 0.395, 1.341, 0.419, 1.608	−1.469, −1.04, −0.217, 0.259, 0.069, 0.486
IGHV4-39	0.134, “NA”, −0.611, “NA”, 0.492, “NA”, 0.21, “NA”, −0.567, “NA”, 0.53, “NA”, 1.584, “NA”, 0.803, “NA”, 1.897, “NA”	“on”, “NA”, −0.251, “NA”, “on”, “NA”, 0.17, “NA”, “NA”, “NA”, “off”, “NA”
DOK1	0.53, 1.243, 0.646, −0.05, 0.633, 0.026, 0.225, 0.901, 0.296	−0.053, −0.727, 0.594, −0.033, −0.302, −0.991
MSANTD3	−0.307, −0.013, −0.307, 0.128, 0.39, 0.089, −0.086, 0.173, −0.13	−0.6, −0.461, −0.24, −0.046, 0.287, 0.423
ZNF137P	−0.714, 0.079, 0.411, 0.024, 0.784, 1.109, −1.273, −0.511, −0.191	−1.909, −0.863, −1.726, −0.628, −0.979, 0.063
HLA-DQA1	“on”, “NA”, “NA”, −0.155, “NA”, “NA”, “on”, “NA”, “NA”, −0.203, “NA”, “NA”, −1.627, “NA”, “NA”, 0.469, “NA”, “NA”, “on”, “NA”, “NA”, −0.746, “NA”, “NA”, “on”, “NA”, “NA”, −0.827, “NA”, “NA”, −1.268, “NA”, “NA”, −0.162, “NA”, “NA”, “NA”, “NA”, “NA”, 0.049, “NA”, “NA”, “NA”, “NA”, “NA”, −0.035, “NA”, “NA”, “off”, “NA”, “NA”, 0.629, “NA”, “NA”	“on”, “NA”, “NA”, 1.95, “NA”, “NA”, “on”, “off”, “NA”, 2.317, “NA”, “NA”, “NA”, “NA”, “NA”, 2.114, “NA”, “NA”, ‘ “NA”, “off”, “NA”, 2.537, “NA”, “NA”, “on”, “on”, “NA”, 2.238, “NA”, “NA”, “on”, 0.227, “NA”, 2.604, “NA”, “NA”
SEPSECS	−0.253, −0.316, 0.132, 0.081, −0.013, 0.427, −0.109, −0.205, 0.231	−0.392, −0.426, −0.137, −0.119, −0.082, −0.122

References

1. Bangsow, T.; Baumann, E.; Bangsow, C.; Jaeger, M.H.; Pelzer, B.; Gruhn, P.; Wolf, S.; von Melchner, H.; Stanimirovic, D.B. The epithelial membrane protein 1 is a novel tight junction protein of the blood-brain barrier. *J. Cereb. Blood Flow Metab.* **2008**, *28*, 1249–1260.
2. Nazli, A.; Chan, O.; Dobson-Belaire, W.N.; Ouellet, M.; Tremblay, M.J.; Gray-Owen, S.D.; Arsenault, A.L.; Kaushic, C. Exposure to HIV-1 directly impairs mucosal epithelial barrier integrity allowing microbial translocation. *PLoS Pathog.* **2010**, *6*, e1000852.
3. Dallasta, L.M.; Pisarov, L.A.; Esplen, J.E.; Werley, J.V.; Moses, A.V.; Nelson, J.A.; Achim, C.L. Blood-brain barrier tight junction disruption in human immunodeficiency virus-1 encephalitis. *Am. J. Pathol.* **1999**, *155*, 1915–1927.
4. Kanmogne, G.D.; Primeaux, C.; Grammas, P. HIV-1 gp120 proteins alter tight junction protein expression and brain endothelial cell permeability: Implications for the pathogenesis of HIV-associated dementia. *J. Neuropathol. Exp. Neurol.* **2005**, *64*, 498–505.
5. Laspiur, J.P.; Anderson, E.R.; Ciborowski, P.; Wojna, V.; Rozek, W.; Duan, F.; Mayo, R.; Rodríguez, E.; Plaud-Valentín, M.; Rodríguez-Orengo, J.; *et al.* CSF proteomic fingerprints for HIV-associated cognitive impairment. *J. Neuroimmunol.* **2008**, *205*, 161–161, doi:10.1016/j.jneuroim.2008.07.009.
6. Garaci, E.; Caroleo, M.C.; Aloe, L.; Aquaro, S.; Piacentini, M.; Costa, N.; Amendola, A.; Micera, A.; Caliò, R.; Perno, C.F.; *et al.* Nerve growth factor is an autocrine factor essential for the survival of macrophages infected with HIV. *Proc. Natl. Acad. Sci. USA* **1999**, *96*, 14013–14018.
7. Schifitto, G.; Yiannoutsos, C.; Simpson, D.M.; Adornato, B.T.; Singer, E.J.; Hollander, H.; Marra, C.M.; Rubin, M.; Cohen, B.A.; Tucker, T.; *et al.* Long-term treatment with recombinant nerve growth factor for HIV-associated sensory neuropathy. *Neurology* **2001**, *57*, 1313–1316.
8. Goletti, D.; Kinter, A.L.; Hardy, E.C.; Poli, G.; Fauci, A.S. Modulation of endogenous IL-1 beta and IL-1 receptor antagonist results in opposing effects on HIV expression in chronically infected monocytic cells. *J. Immunol.* **1996**, *156*, 3501–3508.
9. Schoggins, J.W.; Rice, C.M. Interferon-stimulated genes and their antiviral effector functions. *Curr. Opin. Virol.* **2011**, *1*, 519–525.
10. Fensterl, V.; Wetzel, J.L.; Ramachandran, S.; Ogino, T.; Stohlman, S.A.; Bergmann, C.C.; Diamond, M.S.; Virgin, H.W.; Sen, G.C. Interferon-induced Ifit2/ISG54 protects mice from lethal VSV neuropathogenesis. *PLoS Pathog.* **2012**, *8*, e1002712.
11. Greenwell-Wild, T.; Vázquez, N.; Jin, W.; Rangel, Z.; Munson, P.J.; Wahl, S.M. Interleukin-27 inhibition of HIV-1 involves an intermediate induction of type I interferon. *Blood* **2009**, *114*, 1864–1874.
12. Pertel, T.; Reinhard, C.; Luban, J. Vpx rescues HIV-1 transduction of dendritic cells from the antiviral state established by type 1 interferon. *Retrovirology* **2011**, *8*, doi:10.1186/1742-4690-8-49.
13. Jolly, C.; Mitar, I.; Sattentau, Q.J. Requirement for an intact T-cell actin and tubulin cytoskeleton for efficient assembly and spread of human immunodeficiency virus type 1. *J. Virol.* **2007**, *81*, 5547–5560.
14. Matarrese, P.; Malorni, W. Human immunodeficiency virus (HIV)-1 proteins and cytoskeleton: Partners in viral life and host cell death. *Cell Death Differ.* **2005**, *12*, 932–941.
15. Soto-Giron, M.J.; Garcia-Vallejo, F. Changes in the topology of gene expression networks by human immunodeficiency virus type 1 (HIV-1) integration in macrophages. *Virus Res.* **2012**, *163*, 91–97.
16. Repunte-Canonigo, V.; Lefebvre, C.; George, O.; Kawamura, T.; Morales, M.; Koob, G.F.; Califano, A.; Masliah, E.; Sanna, P.P. Gene expression changes consistent with neuroAIDS and impaired working memory in HIV-1 transgenic rats. *Mol. Neurodegener.* **2014**, *9*, doi:10.1186/1750-1326-9-26.
17. Roshal, M.; Kim, B.; Zhu, Y.H.; Nghiem, P.; Planelles, V. Activation of the ATR-mediated DNA damage response by the HIV-1 viral protein R. *J. Biol. Chem.* **2003**, *278*, 25879–25886.
18. Andersen, J.L.; Planelles, V. The role of Vpr in HIV-1 pathogenesis. *Curr. HIV Res.* **2005**, *3*, 43–51.
19. Montano, M.; Rarick, M.; Sebastiani, P.; Brinkmann, P.; Russell, M.; Navis, A.; Wester, C.; Thior, I.; Essex, M. Gene-expression profiling of HIV-1 infection and perinatal transmission in Botswana. *Genes Immun.* **2006**, *7*, 298–309.

20. Hu, H.; Nau, M.; Ehrenberg, P.; Chenine, A.L.; Macedo, C.; Zhou, Y.; Daye, Z.J.; Wei, Z.; Vahey, M.; Michael, N.L.; *et al.* Distinct gene-expression profiles associated with the susceptibility of pathogen-specific CD4 T cells to HIV-1 infection. *Blood* **2013**, *121*, 1136–1144.
21. Foley, J.F.; Yu, C.R.; Solow, R.; Yacobucci, M.; Peden, K.W.; Farber, J.M. Roles for CXC chemokine ligands 10 and 11 in recruiting CD4(+) T cells to HIV-1-infected monocyte-derived macrophages, dendritic cells, and lymph nodes. *J. Immunol.* **2005**, *174*, 4892–4900.
22. Bell, B.; Sadowski, I. Ras-responsiveness of the HIV-1 LTR requires RBF-1 and RBF-2 binding sites. *Oncogene* **1996**, *13*, 2687–2697.
23. Toschi, E.; Bacigalupo, I.; Strippoli, R.; Chiozzini, C.; Cereseto, A.; Falchi, M.; Nappi, F.; Sgadari, C.; Barillari, G.; Mainiero, F.; *et al.* HIV-1 Tat regulates endothelial cell cycle progression via activation of the Ras/ERK MAPK signaling pathway. *Mol. Biol. Cell* **2006**, **17**, 1985–1994.
24. Manninen, A.; Renkema, G.H.; Saksela, K. Synergistic activation of NFAT by HIV-1 Nef and the Ras/MAPK pathway. *J. Biol. Chem.* **2000**, *275*, 16513–16517.
25. Eugenin, E.A.; Berman, J.W. Gap junctions mediate human immunodeficiency virus-bystander killing in astrocytes. *J. Neurosci.* **2007**, *27*, 12844–12850.
26. Moreno-Fernandez, M.E.; Mauricio Rueda, C.; Rusie, L.K.; Chougnet, C.A. Regulatory T cells control HIV replication in activated T cells through a cAMP-dependent mechanism. *Blood* **2011**, *117*, 5372–5380.
27. Li, M.W.M.; Mruk, D.D.; Cheng, C.Y. Gap junctions and blood-tissue barriers. *Adv. Exp. Med. Biol.* **2012**, *763*, 260–280.
28. Johnston, J.B.; Zhang, K.; Silva, C.; Shalinsky, D.R.; Conant, K.; Ni, W.; Corbett, D.; Yong, V.W.; Power, C. HIV-1 Tat neurotoxicity is prevented by matrix metalloproteinase inhibitors. *Ann. Neurol.* **2001**, *49*, 230–241.
29. Webster, N.L.; Crowe, S.M. Matrix metalloproteinases, their production by monocytes and macrophages and their potential role in HIV-related diseases. *J. Leukoc. Biol.* **2006**, *80*, 1052–1066.
30. Mellanen, L.; Lahdevirta, J.; Tervahartiala, T.; Meurman, J.H.; Sorsa, T. Matrix metalloproteinase-7,-8,-9,-25, and-26 and CD43,-45, and-68 cell-markers in HIV-infected patients' saliva and gingival tissue. *J. Oral Pathol. Med.* **2006**, *35*, 530–539.
31. Schrager, J.A.; Marsh, J.W. HIV-1 Nef increases T cell activation in a stimulus-dependent manner. *Proc. Natl. Acad. Sci. USA* **1999**, *96*, 8167–8172.

© 2015 by the authors; licensee MDPI, Basel, Switzerland. This article is an open access article distributed under the terms and conditions of the Creative Commons by Attribution (CC-BY) license (http://creativecommons.org/licenses/by/4.0/).
